# Plant functional trait responses to cope with drought in seven cool-season grasses

**DOI:** 10.1038/s41598-023-31923-y

**Published:** 2023-03-31

**Authors:** Mohammad Hadi Taleb, Mohammad Mahdi Majidi, Fatemeh Pirnajmedin, Sayed Ali Mohammad Mirmohammady Maibody

**Affiliations:** Department of Agronomy and Plant Breeding, College of Agriculture, Isfahan University of Technology, Isfahan, 84156-83111 Iran

**Keywords:** Plant breeding, Plant ecology, Plant physiology, Plant stress responses

## Abstract

In semi-arid and arid regions, the selection of suitable grass species with high-yield production, tolerance to drought stress, and potential for recovery from drought is of special importance. Despite extensive research in cool-season grasses, inter-species differences in post-drought recovery, persistence, survival, and summer dormancy and their relationship with drought tolerance need more investigation. In the present study, 28 diverse genotypes belonged to seven cool-season grass species, including *Festuca arundinacea* (tall fescue), *Festuca pratensis* (meadow fescue), *Festuca ovina* (sheep fescue), *Festuca rubra* (red fescue), *Lolium perenne* (perennial ryegrass), *Lolium multiflorum* (Italian ryegrass) and *Lolium* × *hybridum* were evaluated during 2016–2019 under three irrigation regimes (normal, mild, and intense drought stress). Then in the fourth year (on August 2019), irrigation was withheld at all previous irrigation regimes for two months during summer, and then species were re-irrigated to study the effect of prolonged drought conditions. A wide range of genetic diversity was detected in all the measured traits among and within species in response to different irrigation levels. Recurrent drought stress decreased forage productivity, post-drought recovery, and survival in all grass species. Among the studied species, tall fescue had higher forage production, drought tolerance, survival, recovery rate, and persistence. Sheep fescue had low forage production and recovery after drought. Drought tolerance (based on stress tolerance score, STS) was highly associated with forage yield and post-drought recovery and partially with summer dormancy under both mild and intense drought stress conditions. This indicated that selection based on higher STS would lead to choosing genotypes with better recovery after prolonged drought. Superior species and preferable genotypes for forage use from species *Festuca arundinacea* and for turf application from species *Festuca arundinacea*, *Lolium perenne* and *Lolium* × *hybridum* were identified across different water environments for future programs.

## Introduction

According to the global climate change scenarios, the frequency and intensity of drought are predicted to increase, and this condition in drought-prone regions is expected to worsen over the next few decades^1^. Conceivably, such changes in water availability in dry areas will likely alter plant community composition and the critical ecosystem functions^2^. Drought events are the major problems worldwide, which has led to a reduction in agricultural productivity, development, and survival^3^. One of the best strategies to prevent the unfavorable effects of water stress is selecting resistant species and genotypes^4^. Traits associated with water in plants reflect evolutionary history, and influence individual performance, plant community composition, and ecosystem function, and offer insight into which genotypes will most likely be affected by changes in water availability. Diversity in different acquisition strategies such as root characteristic system, survival, productivity, persistence, summer dormancy, and post-drought recovery help to identify which species are most vulnerable to drought^5^.

Perennial forage grass species, due to their pivotal role in healthy fodder production for livestock consumption, amenity, and carbon fixation in ecology have a lot of importance among plants^6^. The grass species' response to water deficiency and their ability to forage production during and after drought stress conditions are greatly varied. Perennial forage species due to their ability to offer better water use efficiency with a fast regrowth at the beginning of the fall rains and more efficient use of residual moisture in the soil at the end of spring, and reduction in risk of soil erosion relative compared with annual forage species are preferable and can be a valuable substitute for the predominantly drought-sensitive annual species^7^. Perennial forage grasses species use different mechanisms and characteristics to survive during drought stress conditions, which include dehydration avoidance, dehydration tolerance, escape, and summer dormancy. The expression level of dehydration avoidance and tolerance approaches in plant genotypes is dependent on the plant growth stages, the level of drought stress, and their interaction^8^.

Most perennial grass species have some other specific advantages such as high persistence and rapid recovery after repeated summer drought which can remain in pastures and better compete with other species^9^. Recovery and the regeneration of new tissue when the water supply is available following drought stress are largely dependent on the ability of plants to accumulate biomass through the mechanism of rapid growth^10^. Selection based on the traits related with high survival and recovery after prolonged drought stress condition may be of greater economic importance than just the choice for improved growth during droughts^11^. The post-drought recovery and survival traits lead to better competition for plants with less drought-tolerant species and redound better persistence in pastures^12^. In forage species, favorable genotypes must have not only the potential of high persistence, and survival through repeated summer droughts, but also have favorable forage production^10^. The difference in produced forage across years can provide an idea of persistence, especially in space plant material^13^. In turfgrass species, the potential of high persistence and post-drought recovery is more important than the higher yield production^14^. Mechanisms facilitating the ability of turfgrass to post-drought recovery and persistence have been studied in a few perennial forage species such as *Dactylis glomerata* L.^14,15^ and *Bromus inermis*^16^ however no comprehensive study have been done in important grasses.

Little information is available about the comparison and diversity of different grass species under different moisture regimes for drought tolerance, survival, persistence, summer dormancy, and post-drought recovery-related traits. This study aimed (i) to compare seven common grass species and within species genetic diversity in terms of drought tolerance under different irrigation treatments, (ii) to assess functional traits including survival, persistence, productivity, and summer dormancy during consecutive years and investigate the relationship of these traits with post-drought recovery of genotypes.

## Results

### Analysis of variance and mean comparison of traits

Analysis of variance (ANOVA) indicated significant variability among the genotypes (G) and species (S) for dry forage yield (DFY), survival (SU), recovery rate (RR) traits, and stress tolerance score (STS) (Supplementary Tables S2 and S3). The effects of irrigation treatments (IT), S × IT, and G × IT interaction were also significant for these traits (Supplementary Tables S2 and S3). The effect of the species and genotypes was significant (P < 0.01) for persistence (PE) and summer dormancy (S/SP) under normal irrigation condition (Supplementary Table S4).

### Productivity and trends of growth during years

Mean comparisons of different traits for three moisture environments for species and genotypes are presented in Tables 1 and 2, respectively. The results of the dry forage yield (DFY) under three moisture environments revealed high genetic variation between various species (Table 1). Mild drought stress condition led to the significant reduction in DFY in all species except *Festuca pratensis.* Intense drought stress condition also led to the considerable decrease in DFY in all species compared with normal irrigation level (Table 1). The highest value of DFY was observed in *Festuca arundinacea* species, and the lowest values of this trait were obtained for *Festuca ovina* and *Festuca rubra* species in all the moisture environments (Table 1). The highest reduction in DFY was recorded for *Festuca ovina* (50%), and the lowest for *Festuca pratensis* (29%) in mild drought stress, and the highest decrease was observed for *Lolium perenne* (77%) and the lowest for *Lolium* × *hybridum* (61%) in intense drought stress (Table 1). Mean comparisons of genotypes for DFY were presented in Table 2. Genotype Fa1E had the greatest value of DFY under normal irrigation, and genotype FaFe had the greatest value of DFY under both drought stress conditions. Genotypes FrRu2, FrRu4, and FrRu5 under normal irrigation treatment and genotypes FrRu1, FrRu2, FrRu3, FrRu4, FrRu5, and FoOv132 under mild drought stress and genotypes FrRu1, FrRu2, FrRu3, FrRu4, FrRu5, FrRu6, FoOv132, LpArv, and LmAl under intense drought stress showed the lowest DFY (Table 2).

**Table 1:** Means of measured traits in seven grass species under three irrigation treatments (normal, mild and intense drought stress).

Species	DFY (g/plant)			SU (%)			RR (1–9)			STS		S/SP	PE
	Normal	Mild stress	Intense stress	Normal	Mild stress	Intense stress	Normal	Mild stress	Intense stress	Mild stress	Intense stress	Normal	Normal
*Festuca arundinacea*	568.8a	308.0b	163.0c	77.0a	68.2b	59.7c	6.4a	5.1b	3.5c	13.3a	14.2a	5.87	152.2
*Festuca pratensis*	260.5a	183.8ab	70.5b	15.2a	13.2ab	11.0b	6.3a	4.7ab	3b	2.3a	− 24.8b	5.63	− 93.7
*Festuca ovina*	129.3a	64.3b	42.8c	47.5a	36.4b	35.1b	4.7a	3.1b	2.5b	− 27.1b	− 17.6a	6.03	19.2
*Festuca rubra*	98.7a	63.4b	36.9c	51.7a	39.2b	33.0c	3.2a	2.8ab	2.4b	− 14.4b	− 6.2a	4.99	− 16.1
*Lolium perenne*	205.7a	113.8b	48.3c	50.0a	48.5a	39.4b	4.8a	3.1b	2.6b	− 13.7a	− 19.9b	5.51	− 120.4
*Lolium* × *hybridum*	210.2a	139.0b	81.0c	47.6a	46.7b	38.2c	4.7a	3.6ab	2.7b	− 3.2b	6.4a	5.87	− 123.1
*Lolium multiflorum*	158.1a	101.1b	43.5c	16.7a	15.3a	12.4b	4.3a	3.6a	2.2b	13.3a	0.1b	3.73	− 186.8
LSD_0.05_	38.3	38.4	23.8	1.9	4.0	4.0	0.5	0.3	0.5	1.7	1.5	0.81	6.2

*DFY* dry forage yield, *SU* survival, *RR* recovery rate, *STS* stress tolerance score, *S/SP* summer dormancy, *PE* persistence.

In each row (among three irrigation environments) means followed by a common letter are not significantly different according to the LSD test at an alpha level of 0.05.

In each column, If the difference between the means is greater than the LSD_0.05_, then the means (among species) are significantly different.

**Table 2:** Means of measured traits in 28 genotypes of various grass species under normal, mild and intense drought stress.

Species	Genotype	DFY (g/plant)			SU (%)			RR (1–9)			STS		S/SP	PE
		Normal	Mild stress	Intense stress	Normal	Mild stress	Intense stress	Normal	Mild stress	Intense stress	Mild stress	Intense stress	Normal	Normal
*Festuca arundinacea*	Fa1E	999.3a	289.2b	143.6b	90.0a	85.2a	69.2b	6.5a	5.8a	3.2b	− 18.11a	− 21.8a	5.35	495.1
	Fa21M	703.2a	355.9b	162.3c	85.0a	72.7b	69.5b	6.6a	5.4a	3.3b	17.2a	4.6b	5.24	340.1
	Fa17M	357.1a	319.7a	189.1b	80.0a	73.0ab	70.9b	4.5a	4.7a	3.5b	37.1b	44.6a	4.99	173.6
	Fa4E	878.6a	318.3b	121.9b	95.0a	81.6b	69.2c	7.6a	5.1b	2.6c	− 3.6a	− 25.6b	4.91	463.6
	FaFe	567.3ab	700.7a	354.3b	100.0a	86.4b	73.2c	7.7a	7.9a	5.4b	129.8a	112.6a	6.61	91.2
	FaLu	584.8a	524.1b	274.3c	90.0a	81.1a	66.2b	6.8a	6.2a	4.7b	75.4b	69.0a	6.16	− 6.2
	FaBe	248.8a	149.1b	85.3b	50.0a	53.5a	41.4b	4.1a	4.4a	2.7b	− 8.9b	− 2.4a	7.80	− 197.6
	FaMo	268.6a	99.7b	125.2b	35.0a	30.0b	24.4c	4.0a	3.9a	3.7a	− 30.8b	16.4a	7.80	− 120.5
	FaEl	637.6a	197.1b	73.2c	70.0a	58.1b	56.7b	8.0a	4.2b	3.1b	− 27.4a	− 39.8a	5.43	212.2
	FaBa	442.5a	126.5b	100.4b	75.0a	60.5b	55.5b	8.3a	3.6b	3.3b	− 37.0b	− 15.2a	4.41	70.3
*Festuca pratensis*	FpPre	303.0a	243.7a	89.1b	14.0a	12.5ab	10.5b	7.4a	5.7ab	3.4b	20.0a	− 3.5b	5.95	− 78.1
	FpPra	218.0a	123.7ab	51.8b	16.5a	14.0b	11.5c	5.2a	3.7ab	2.5b	− 15.3a	− 46.0b	5.31	− 149.4
*Festuca ovina*	FoOv69	142.2a	67.5b	47.2c	50.0a	35.2b	34.7b	4.6a	3.0b	2.7b	− 28.9b	− 10.8a	6.34	23.5
	FoOv132	116.3a	61.1b	38.3b	45.0a	38.0b	34.8b	4.9a	3.2b	2.4c	− 25.3a	− 24.4a	5.72	15.0
*Festuca rubra*	FrRu1	90.8a	55.2b	39.4c	70.0a	43.5b	40.0b	3.2a	2.6a	3.1a	− 19.8b	− 4.4a	6.74	9.9
	FrRu2	55.6a	43.6ab	39.6b	55.0a	39.7b	35.5b	3.5a	2.8a	2.4a	4.8b	18.3a	4.39	− 19.9
	FrRu3	110.8a	55.7a	36.0c	55.0a	43.2b	36.7b	3.0a	2.2b	2.1b	− 27.5b	− 15.0a	3.26	− 35.0
	FrRu4	79.0a	60.4b	38.0c	40.0a	27.8b	22.8c	2.4b	3.2a	2.1b	− 11.6b	− 1.8a	5.38	− 17.5
	FrRu5	89.2a	57.0b	36.6b	50.0a	46.0a	36.3b	2.6a	2.3a	2.6b	− 21.2b	− 6.5a	5.30	− 19.4
	FrRu6	166.5a	108.4ab	31.5b	40.0a	35.2ab	26.7b	4.5a	3.8ab	2.1b	− 11.3a	− 27.7b	4.85	3.4
*Lolium perenne*	LpArv	258.9a	79.1b	23.9c	65.0a	51.8b	46.8b	5.0a	2.4b	2.1b	− 38.2a	− 43.4b	5.50	− 198.8
	LpAri	152.4a	148.5a	72.8b	35.0b	45.3a	32.0b	4.7a	3.8ab	3.1b	10.8a	3.6b	5.52	− 42.0
*Lolium* × *hybridum*	LhRu	360.7a	184.4b	95.6b	45.0ab	51.8a	38.0c	5.9a	4.0ab	2.8b	− 13.1a	− 9.6a	5.65	− 73.9
	LhRe	105.7a	78.0ab	62.3b	55.0a	40.3b	38.9c	3.7a	3.0ab	2.6b	− 9.8b	13.6a	6.30	− 162.2
	LhTa	164.6a	154.6a	84.9b	40.0b	51.0a	37.9b	4.5a	3.9a	2.9c	13.2b	15.2a	5.68	− 133.3
*Lolium multiflorum*	LmAl	230.2a	122.7b	32.5c	22.5a	11.3b	8.0b	4.6a	4.0a	2.2b	20.9a	− 24.7b	5.47	− 111.9
	LmAx	132.5a	99.7a	48.3b	15.0a	12.5b	17.3a	4.5a	3.9a	2.3b	− 8.4a	− 13.6b	2.73	− 238.8
	LmOr	111.7a	81.0a	49.8b	12.5b	22.2a	12.0b	3.8a	2.9ab	2.3b	27.4b	38.7a	6.40	− 209.6
	LSD_0.05_	74.2	56.9	42.1	3.4	6.2	5.2	0.8	0.8	0.7	2.9	2.9	1.75	1.0

*DFY* dry forage yield, *SU* survival, *RR* recovery rate, *STS* stress tolerance score, *S/SP* summer dormancy, *PE* persistence.

In each row, (among three irrigation environments) means followed by a common letter are not significantly different according to the LSD test at an alpha level of 0.05.

In each column, If the difference between the means is greater than the LSD_0.05_, then the means (among genotypes) are significantly different.

For information about plant materials and their origins see Table S1.

**Figure 1:**
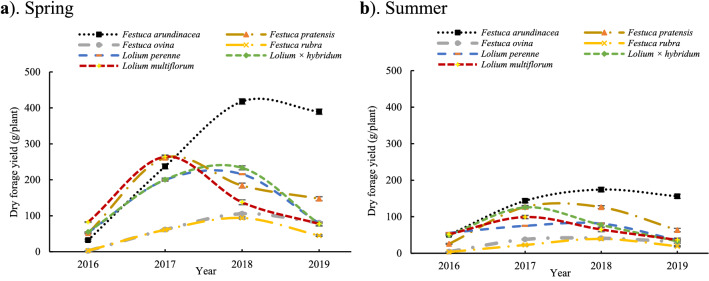
Trends of spring and summer dry forage yield (DFY) over years (2016–2019) in seven grass species subjected to normal irrigation condition. Error bar in each point is standard error in every year.

Dry forage yield (DFY) of spring and summer cuts for seven species under normal irrigation condition over the years (2016–2019) is presented in Fig. 1. In general, after the first year (establishment year), the forage production trend showed that the spring forage production was more than the summer forage yield in all species (Fig. 1). Yield trends were different for each species during four years, for example *Lolium multiflorum* and *Festuca pratensis* species revealed the highest production in the second year, while for the other species turning point happened in the third year (Fig. 1). Trends of DFY during four years in spring cut showed that some species have similar growth trends. The growth trends for the two species of *Lolium multiflorum* and *Festuca pratensis* was similar, so that they showed the highest yield in the second year and this trend decreased in the next years. Two species of *Festuca rubra* and *Festuca ovina* had similar growth behavior during the growth trend and had lower yield than other species. The growth trends for species *Lolium perenne* and *Lolium* × *hybridum* were also similar. The trend of growth in *Festuca arundinacea* was clearly different from other species as had the highest amount of yield in the third and fourth years (Fig. 1).

### Drought stress tolerance

The mean comparison of species for stress tolerance score (STS) (Table 1) revealed that *Festuca arundinacea* and *Lolium multiflorum* had the highest STS under mild drought stress condition, while under intense drought stress *Festuca arundinacea* and then *Lolium* × *hybridum* were superior in terms of STS. Between the studied genotypes large genetic variation was found for STS (Table 2). Under mild and intense drought stress conditions, genotypes FaFe and LpArv had higher and lower STS, respectively (Table 2).

### Persistence of perennial cool‐season grasses

The mean comparison of persistence (PE) for seven species under normal irrigation condition is shown in Table 1. The PE had extensive variation in the seven species, so that the PE ranged from − 186.8 to 152.2. Under normal irrigation condition, *Festuca arundinacea* and *Lolium multiflorum* had the highest and lowest value of the PE, respectively (Table 1). The *Festuca arundinacea* and *Festuca ovina* species showed positive PE, and other species showed negative PE. Mean comparison of genotypes revealed that Fa1E had the highest value of PE and LmAx had the lowest value of this trait (Table 2).

**Figure 2:**
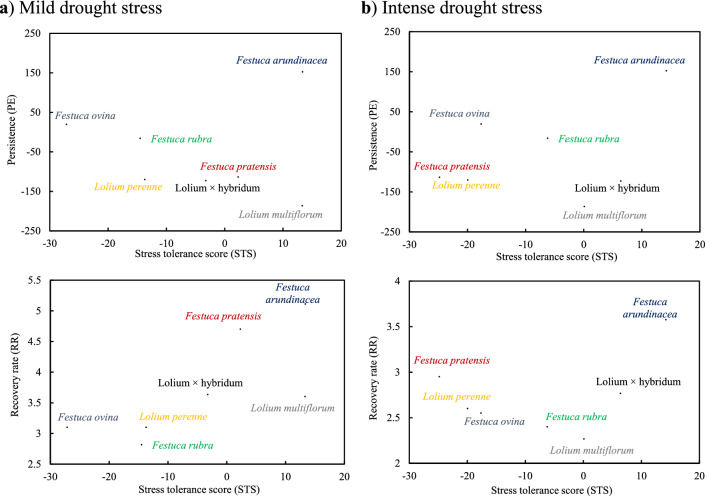
Biplot of stress tolerance score (STS) vs. persistence (PE) and recovery rate (RR) for seven grass species under two drought stress levels (mild and intense drought stress).

Biplots of persistence (PE) vs. stress tolerance score (STS) for seven species and 28 genotypes under two drought stress levels (mild and intense drought stress) are shown in Fig. 2 and Supplementary Fig. S1, respectively. Under both mild and intense drought stress conditions, *Festuca arundinacea* had the highest value of both STS and PE, while *Lolium perenne* had the lowest values of these traits (Fig. 2). In the biplots for genotypes under mild and intense drought stress conditions, the highest value of STS and moderate values of PE was obtained for genotypes FaFe, and FaLu and the lowest value were detected for genotypes FaBe, LmAx, FrRu3, FrRu5 and LpArv (Supplementary Fig. S1).

### Survival of grasses

Intense drought stress significantly decreased SU in all species, while mild drought stress reduced SU in some species (*Festuca arundinacea*, *Festuca ovina*, and *Festuca rubra*) (Table 1). The average reduction of SU for *Festuca arundinacea* was 11% and 22% and for *Festuca rubra* was 24% and 36% under mild and intense drought stress conditions, respectively (Table 1). Great genetic diversity was shown among genotypes for SU under normal and drought stress conditions (Table 2). Genotype FaFe from *Festuca arundinacea* had the highest values of SU among all the evaluated genotypes at three irrigation treatments (Table 2). Overall, genotypes FpPre and FpPra from *Festuca pratensis* species and genotypes LmAl, LmAx, and LmOr from *Lolium multiflorum* species had lower values of SU than the other genotypes at three irrigation treatments (Table 2).

**Figure 3:**
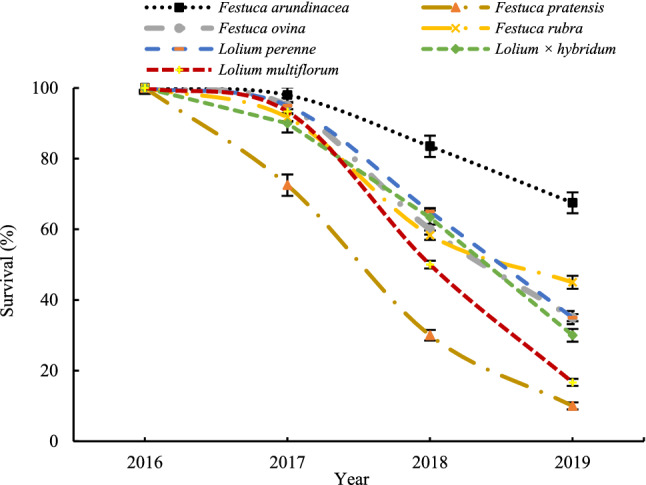
Trends of survival in seven grass species subjected to normal irrigation condition over years (2016–2019). Error bar in each point is standard error in every year.

The survival trend for all species under normal irrigation condition revealed that this trait was significantly reduced from the second year to the fourth year in all species (Fig. 3). The highest and lowest survival values were related to the *Festuca arundinacea* and *Festuca pratensis* species, respectively (Fig. 3).

### Summer dormancy in perennial cool-season grasses

The mean comparison of species revealed that *Festuca arundinacea* and *Festuca ovina* had the highest while *Lolium multiflorum* species had the lowest value of summer dormancy (Table 1). The mean comparison of S/SP for the 28 genotypes under normal irrigation condition (Table 2) indicated that a large genetic variation was observed among the genotypes for this trait (Table 2). Genotypes FaBe and FaMo displayed the highest value of S/SP, and genotypes LmAx, FaBa, FrRu2, and FrRu3 revealed the low values of S/SP (Table 2).

### Recovery after long drought stress

The results of mean comparisons of irrigation treatments for the recovery rate (RR) in the fourth year (2019), after stopping irrigation for two months and then re-watering, showed that intense drought stress reduced RR in all of the species. Results revealed that the RR decreased by 24% and 45% under prolonged mild and intense drought stress conditions compared to normal irrigation, respectively. Mild drought stress didn’t affect on RR of four species of *Festuca pratensis*, *Festuca rubra*, *Lolium multiflorum*, and *Lolium* × *hybridum* (Table 1). The range of RR in all species under intense drought stress was varied from 3.5 (*Festuca arundinacea*) to 2.2 (*Lolium multiflorum*) (Table 1). The range of RR reduction for *Festuca arundinacea* was 20% and 44% and for *Lolium multiflorum* was 16% and 47% under mild and intense drought stress conditions, respectively (Table 1). This range was from 5.1 (*Festuca arundinacea*) to 2.8 (*Festuca rubra*) under mild and from 6.4 (*Festuca arundinacea*) to 3.2 (*Festuca rubra*) under normal irrigation conditions. Mean comparisons of all studied genotypes for the RR trait at three irrigation treatments (IT) are shown in Table 2. Under normal irrigation condition, genotypes FaFe, FaBa and FaEl and under drought stress conditions, genotype FaFe had higher RR. Meanwhile, genotypes FrRu3, FrRu4, and FrRu5 had the lowest values of RR under three IT (Table 2).

Biplots of recovery rate (RR) vs. stress tolerance score (STS) for seven species and 28 genotypes under mild and intense drought treatments (Fig. 2 and Supplementary Fig. S1) showed that simultaneous selection for increasing both RR and STS might be possible among the studied grass species. For example, according to the biplot, *Festuca arundinacea* and *Festuca pratensis* had the highest values of STS and RR traits under mild drought stress condition, while under intense drought stress, only *Festuca arundinacea* was superior in terms of both STS and RR (Fig. 2). Among the studied genotypes, FaFe and FaLu revealed high values of STS and RR traits under both mild and intense drought stress conditions (Supplementary Fig. S1). Meanwhile, *Lolium perenne*, *Festuca ovina*, and *Festuca rubra* species and LpArv, FoOv69, FrRu3, and FrRu5 genotypes had the lowest values of STS and RR traits under both mild and intense drought stress conditions (Fig. 3 and Supplementary Fig. S1).

### Relationships among traits and selection based on multiple traits

**Figure 4:**
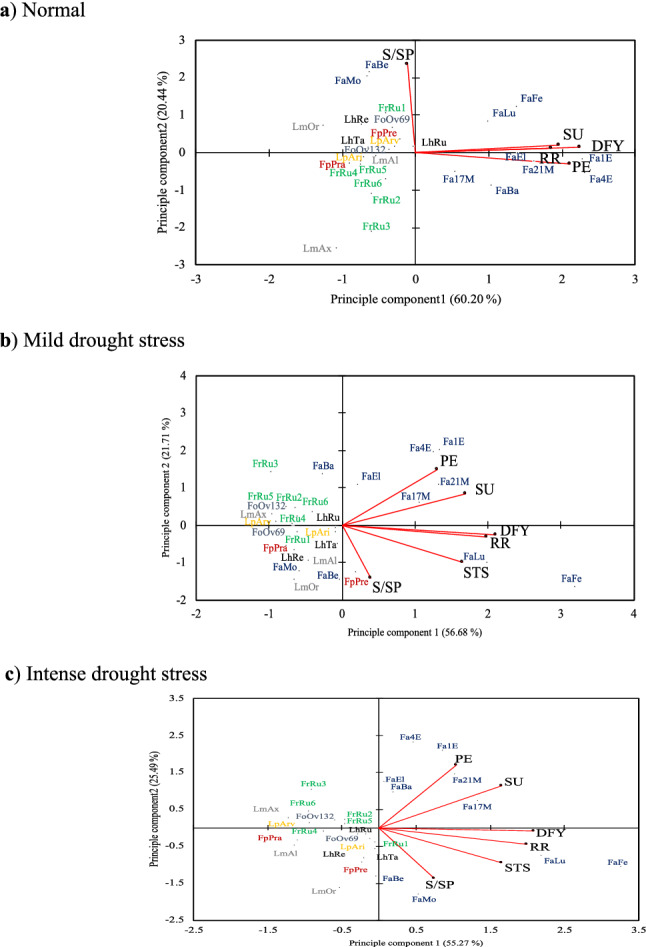
Biplot display of important traits of 28 genotypes of different grasses under three irrigation levels (normal, mild and intense drought stress). *DFY* dry forage yield, *PE* persistence, *RR* recovery rate, *S/SP* summer dormancy, *STS* stress tolerance score, *SU* survival.

In the present study, the relationship between traits and the selection of genotypes based on the multiple traits was done by the principal component analysis (PCA) (Fig. 4). PCA is a practical and standard tool for the dimensional decrease of a set of some observations, each with many variables, and it makes connections between different datasets. In the PCA biplot, the extent of correlation between traits is shown by the cosine of the angle between vectors (< 90° displays positive correlations and 90° < shows a negative correlation). Furthermore, the length of vectors connecting traits to the origin shows the extent of variability and contribution of each trait.

Results of the PCA based on dry forage yield (DFY), recovery rate (RR), persistence (PE), summer dormancy (S/SP), survival (SU), and stress tolerance score (STS) under three irrigation treatments are given in Fig. 4. The PCA results justified that the two main principal components (PC1 and PC2) described approximately more than 81%, 78%, and 81% of the total genetic variation under normal, mild, and intense drought stress conditions, respectively (Fig. 4). The angle between vectors clearly showed that DFY, RR, and STS were highly correlated under three irrigation treatments (Fig. 4). No association was found between PE and S/SP. Under the normal irrigation condition, there were two distinct groups of traits. The first group is SU, DFY, RR, and PE, which are highly associated with PC1. The second group included only S/SP, which is highly associated with PC2. Therefore, under normal condition, selection based on high PC1 and PC2 would lead to selection genotypes with preferable dry matter production, high persistence, recovery after drought and summer dormancy. In this case, genotypes FaFe, FaLu and Fa1E were identified as the superior genotypes (Fig. 4a).

Under mild and intense drought stress conditions (Fig. 4b) the association of traits was considerably changed. Under these conditions, DFY and RR are still highly correlated, while the PE and SU vectors were moved somewhat further. If the purpose is the selection of genotypes for persistence and survival then Fa1E and Fa21M should be selected. However, if the goal is improving productivity, drought tolerance, and recovery after long drought then FaFe and FaLu are suitable genotypes. In contrast, genotypes LmAx, LmAl and FpPra were identified as weak genotypes with low yield stability, forage production, drought tolerance, and survival under intense drought stress condition (Fig. 4c).

## Discussion

High genetic variation was observed among and within cool-season grass species from two important genera of *Festuca* and *Lolium* in terms of forage production, survival, persistence, summer dormancy, post-drought recovery, and drought tolerance. This variation facilitate the possibility of selecting more desirable species and superior genotypes for future studies. Although the values of measured traits related to function varied among the grasses, but our findings indicated interesting associations between some traits under different drought conditions. Also, responses to cope with drought stress in grasses are changed with differences in moisture environments.

In general, mild and intense drought stress environments lead to 40% and 70% reduction in forage yield, respectively. Similar to our results, some previous studies have reported a decrease in forage or biomass production in *Festuca arundinacea*^17^, *Festuca ovina*^18^, *Lolium multiflorum*^19^ and *Festuca pratensis* and *Lolium perenne*^20^ under drought stress conditions. Drought stress leads to reduction in the soil moisture content and water potential in aerial parts of the plant such as stems and leaves. Low water availability in the root zone hampers plant growth, quality and performance^21^. Grass species of the genus *Festuca* display more yield production and higher levels of drought stress tolerance than the genus *Lolium*^22^. In our study, the genus *Festuca* had higher DFY than the genus *Lolium* in all drought stress conditions. On the other hand, fine fescue had the lowest DFY compared to coarse fescue in all irrigation regimes. Fine fescues (including *Festuca ovina* and *Festuca rubra*) and coarse fescue (including *Festuca arundinacea* and *Festuca pratensis*) are two groups of cool-season grasses used for turf and forage production, respectively^23,24^. Interspecific hybridization between various species of fine fescues may create variability with desirable characters from each of the parental species^23^. The results demonstrated that *Lolium* × *hybridum* had higher yield than *Lolium perenne* and *Lolium multiflorum* species under all three moisture environments. Since this species is obtained from the cross between *Lolium perenne* and *Lolium multiflorum* species, it can be concluded that this cross between different species has increased the forage yield in *Lolium* × *hybridum*. Genetic improvement of *Lolium* × *hybridum* has resulted in the development of more forage grasses with increased stress tolerance. Species *Festuca arundinacea* had the highest forage yield among all the studied species under three irrigation environments, which could be due to its high ploidy level (hexaploid) and extensive and deeper root system^18^. The amount of DFY for *Festuca pratensis* in all irrigation conditions (IT) was lower than *Festuca arundinacea* and higher than other species, which may be due to the fact that this species is one of the ancestors of *Festuca arundinacea*^25^. Tall fescue is a hexaploid (2n = 6x = 42) consisting of three genomes (PPG_1_G_1_G_2_G_2_) with the P genome derived from diploid *Festuca pratensis* (2n = 2x = 14) and the G_1_G_2_ genome from the tetraploid *Festuca arundinacea* var glaucescens (2n = 4x = 28)^26^. It also may be due to the placement of some yield and drought tolerance-related alleles on the PP genome.

Different models based on the behavior of plants under normal and drought stress conditions have been suggested to select superior genotypes^27^. The tolerance and susceptibility indices (STI, SSI, TOL, and DSI) have been widely used for selecting superior genotypes under drought stress conditions, but using an index of stress tolerance score (STS) that has all of the tolerance and susceptibility indices would be more effective^28,29^. Since the STS is calculated from other tolerance/susceptibility indices which each calculated based on the mathematical function of forage yield, it is usual that STS has correlation with forage production. Our results indicated that the STS index could be used to differentiate genotypes under different irrigation levels. Under intense drought stress condition, *Festuca arundinacea* had the highest and *Festuca pratensis* species had the lowest STS. Under mild drought stress condition, the results showed that *Festuca arundinacea* and *Lolium multiflorum* had the highest STS. Therefore, these species can be used in environments with mild drought such as semiarid regions.

Total mortality was not observed in each grass species due to drought stress. However, high genetic diversity was seen among genotypes under normal and drought stress conditions. *Festuca arundinacea* had the highest values of survival at three irrigation treatments. The trends of survival during years indicated that the value of this trait for *Festuca pratensis* and *Lolium multiflorum* was rapidly reduced from the first to the fourth year. Plant responses facilitating survival under severe drought condition, which are mainly associated with both dehydration avoidance and tolerance primarily occurs in meristematic tissues, as these may be the sole plant organs surviving severe drought^30^. Pérez-Ramos et al.^31^ reported that the plant’s ability to survive under drought stress is commonly associated with tolerance, protection and repair mechanisms that preserve the structural integrity of cell membranes in meristematic tissues in grasses. Plants with high leaf dehydration tolerance typically maintain growth for longer into a dry period and so are better able to utilize incident rainfall for growth if and when it occurs^30^.

Persistence (PE) results from adaptation to abiotic (frequent droughts) and biotic stresses in some perennial grasses that leads to yield preservation during the post-cultivation years^32^. Our findings indicated the value of PE for the *Festuca* genus was much higher than the *Lolium* genus. *Festuca arundinacea* had the highest and *Lolium multiflorum* and *Festuca pratensis* had the lowest value of PE and SU, respectively. High PE in *Festuca arundinacea* may be due to the deeper root system and greater ability to store carbohydrates in the crown of this genus. Pirnajmedin et al.^17^ reported that tall fescue may respond to drought through an extensive root system, availability of root distribution in deeper soil layers, maintenance of root growth, and reducing shoot growth.

A moderate relationship was found between dry forage yield (DFY), survival (SU), and PE, which indicates that genotypes with higher forage yield maintain yield during the post-planting years and have higher SU. The associations among traits may be used for indirect improvement in grass breeding. Consistent with our findings, Cullen et al.^33^ reported that PE was closely related to survival. The results of this study revealed that genotypes Fa1E, Fa21M, and Fa17M (from *Festuca arundinacea*) were more persistent and drought-tolerant compared to the other species under drought stress conditions. The present findings indicated that fine fescue and *Lolium* genus had lower growth and forage production and so may be used as turf application. Genotypes FrRu4, FrRu2 (from *Festuca rubra*) and LpAri (from *Lolium perenne*), and LhRe (from *Lolium* × *hybridum*) had more drought-tolerant and persistent than other genotypes which can be used for this purpose.

Summer dormancy (S/SP) is acknowledged as one of the avoidance mechanisms in some perennial cool-season grasses, which enables plant survival by reducing water loss and reallocation of energy and storage in meristems to keep the plant alive and to support regrowth^34^. High genetic variation was observed between and within species for S/SP. The range of the S/SP index indicated that there is incomplete or partial summer dormancy in the evaluated germplasm. Consistent with our results, Norton et al.^35^ and Reed et al.^36^ reported partial S/SP in *Festuca arundinacea*. Other studies have addressed summer dormancy in *Kentucky bluegrass*^37–39^ and *Perennial ryegrass*^40^. It also reported that some cool-season grass species from the Mediterranean basin such as *Festuca arundinacea* Schreb.^35^, *Dactylis glomerata* L.^41^, and *Arum palaestinum*^42^ display partial S/SP with greater growth during the fall season. Norton et al.^30^ reported that perennial grass species with summer dormancy during the summer season will have less yield production in the summer but have more persistence and recovery potential through periods of extended hot and dry conditions. Saving water in summer by reducing forage production may be a dormancy strategy in cool-season turf grasses^43^. Therefore, for the development of turf varieties in semi-arid environments with high temperatures in summer, genotypes with high levels of summer dormancy, low forage production, high extensive root system, and survival would be suitable. Results of the PCA revealed that S/SP had moderate positive correlation with drought tolerance (STS) under intense drought stress condition. Therefore, in arid regions, it is preferred to select cool-season grass with incomplete summer dormancy and higher drought tolerance. Large range of variability for the two traits suggests it may be important to understand different drought tolerance strategies in these grasses.

Major factors underlying the ability of regrowth and rehydration of drought-damaged leaves in perennial grass species are largely unknown. Perennial grasses response differently to survive and grow during periods of limited soil moisture and to recover from drought damage upon re-watering or rainfall events. Recovery rate depends on experienced stress level, weather conditions, soil texture and plant species^1^. In the present study, prolonged drought stress during consecutive years led to the reduction of recovery rate in the seven species of grasses. Also, there was a significant difference between all species and their genotypes for recovery rate (RR), which emphasized the high potential for selection genotypes within our germplasm. The superiority of *Festuca arundinacea* and *Festuca pratensis* species over the other cool-season grass species for RR in all irrigation regimes may be related to greater rhizome in length and volume, which would serve as soluble carbohydrate storage organ during stress. The accumulation of soluble carbohydrates in grass crowns or rhizomes is important for regenerating new shoots and roots^44^. The positive relationship between STS and RR under drought stress conditions showed that selection based on higher STS may lead to genotypes with higher RR and drought tolerance. Similar to our findings, positive relationships have been reported between recovery rate and drought tolerance index in tall fescue under drought stress conditions^45^. Yield stability and the high recovery rate after long drought can be important goals in the breeding programs in perennial grasses under drought stress environments^11^.

In conclusion, different cool-season grass species and their genotypes displayed different abilities for forage production, survival, summer dormancy, persistence, and recovery rate under three irrigation treatments during four years. The results suggest the existence of a strong diversification of strategies among grasses to deal with different recurrent water deficit or to recover after long drought stress. These abilities indicate the extent of genetic variation and high potential of the studied germplasm for introducing suitable grass species for semi-arid and arid regions and developing new varieties for special breeding purposes. Drought stress conditions lead to decreased dry forage yield (DFY), survival (SU), and recovery rate (RR) of evaluated species and their genotypes. Persistence (PE) was positively correlated with SU and DFY in all irrigation environments. This result indicates that genotypes with high recovery potential had high forage production and drought tolerance based on STS. Overall genotypes FaFe, Fa21M, Fa17M, FaLu, Fa4E, and Fa1E had high forage yield, persistence, survival, and recovery rate, which can be suitable for forage use. But genotypes FaBe, FaMo, LhTa, and LpAri with low forage production and high summer dormancy, appropriate persistence and acceptable drought tolerance could be suitable for turf application in semi-arid areas. However, further experiments would be needed to assess turf traits and their association with persistence and post-drought recovery in the evaluated germplasm.

## Materials and methods

### Plant materials and experimental site

In this study, 28 genotypes belonging to seven perennial grass species (*Festuca arundinacea*, *Festuca pratensis*, *Festuca ovina*, *Festuca rubra*, *Lolium perenne*, *Lolium multiflorum*, and *Lolium* × *hybridum*) were used (Supplementary Table S1). The seeds of 12 genotypes were gathered from different regions of Iran. The rest of the genotypes were provided from the Institute of Agroscope, Switzerland (13 genotypes), INRA institute, France (2 genotypes), and the company of Barenbrug, Poland (1 genotype) (Supplementary Table S1). Our plant material is a public panel and comply with relevant institutional, national, and international guidelines and legislation.

This study was conducted at the college of agriculture research farm of the Isfahan University of Technology, located in Lavark, Najaf-Abad, Iran (32° 38′ N, 51° 39′ E, 1627 m asl) during 2016–2019. Based on the 40 years meteorological data, this area receives a mean annual precipitation and temperature of 125 mm and 17 °C, respectively. In this region, there is no rain in the summer from early June to mid-October, and crops must be irrigated during these months. The climate is low semi-desertic and subtropical steppe, according to the Gaussen and Koppen climate classification systems, respectively^46^. The soil at the field test site was a silty clay loam Typic Haplargid (pH 7.5) with an average bulk density of 1.4 g/cm^3^.

### Field evaluation

**Figure 5:**
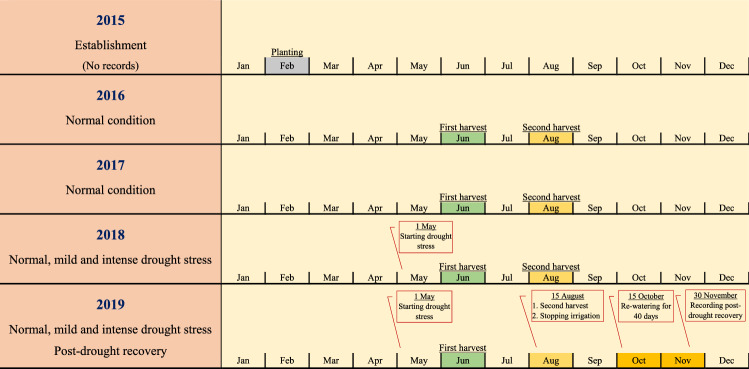
Timing and duration of activities (planting, harvesting and post-drought recovery evaluations) in seven grass species.

The seedlings of each genotype were grown in the greenhouse for three months and then transferred to the field in early February 2015 according to the randomized complete block design with nine blocks (three for each water environment). The three moisture environments are close to each other in the field (2 m distance between environments) and statistical analysis was performed based on combined analysis. In each moisture environment a balanced RCBD design with three blocks was used and in each block all genotypes were randomized. Each plot (genotype) contained 20 individual plants planted in two rows. The distance between rows was 50 cm, and plants within a row were also 50 cm apart. During the first two years after planting (2016–2017), the genotypes were investigated under normal irrigation condition, and during the next 2 years (2018–2019), they were assessed under three irrigation environments (normal, mild, and intense drought stress). The timing and period of establishment and growth and the start of irrigation environments for all species and their genotypes during the studied years are schematically displayed in Fig. 5. In the last year of the experiment (2019), genotypes were assessed for post-drought recovery trait (Fig. 5) which will explain in the next section.

**Figure 6:**
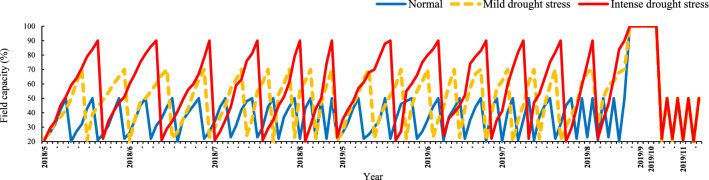
Schematic scheme of percentage of soil water depletion for three different moisture environments (normal, mild and intense drought stress) used in this study. Blue lines denote to the normal environment and orange lines denote to the mild drought stress and red lines denote to the intense drought stress during 2018–2019 (from the first of May to the middle of August). In this study, twenty-eight genotypes (from seven species of cool-season grass) were evaluated for plant functional traits and survival in the field during 2016–2019, under normal and drought stress conditions. In the fourth year (on August 2019), irrigation was withheld in three previous moisture environments for 2 months, and then, plants re-watered (almost 40 days) to detect the effects of prolonged drought stress on post-drought recovery.

Under the normal irrigated level, irrigation was conducted with no limitation and supplied when 50% of the total available water was depleted from the root zone. Under mild and intense drought stresses, irrigation was carried out when 70% and 90% of the total available water was exhausted from the root zone, respectively^47^. In each year, the irrigation treatments were continuously applied throughout the duration of the growing season, from the first of May to the middle of August (Fig. 6). Depending on the weather conditions, intervals of irrigation throughout the course of the growing season and within and among the three water environments were varied (for the normal environment 5–8 days and for mild and intense drought stress environments 8–15 and 15–20 days, respectively). Soil samples were taken every two days from different sites of each irrigation treatment at three depths (0–20, 20–40, and 40–60 cm) to determine the gravimetric soil–water content and irrigation times. For this purpose, we used an auger to make the appropriate-sized hole in the field and took the soil samples. Then the soil samples were dried in an oven for 48 h at 70 °C to calculate the percentage of soil moisture. The irrigation depth was determined according to the following equation:

1 $$I=({\theta}_{FC}-\theta)\times D\times \left(\frac{{\uprho {\text{b}}}}{{\uprho {\text{w}}}}\right)$$

where I, θ_FC_, θ, D, ρb and ρw were irrigation depth (cm), soil gravimetric moisture percent at field capacity, soil gravimetric moisture percentage at irrigating time, the root-zone depth (60 cm), the soil bulk density at root-zone (1.4 g/cm^3^) and water density, respectively. Water was applied using a drip irrigation system through a pumping station, polyethylene pipe, and drip tapes. The applied water volume for each treatment was measured using a volumetric counter.

In each growing year, the aboveground biomass (forage) of genotypes was harvested two times. The forage of all plots was harvested manually from 5 cm above the soil surface, dried at 70 °C for 48 h, and then dry forage yield (DFY) per plant was recorded (total yield produced in spring and summer)^13^. The first harvest was in the late spring (15 June) after flowering and the second one was in the mid-summer (15 August). Summer dormancy (S/SP), survival (SU), and persistence (PE) were measured as recommended by Saeidnia et al.^13^ in late spring. The survival rate (SU) for each genotype was measured based on the percentage of survived plants in the fourth year to the total number of plants after establishment in each genotype in the first year^11^. The persistence (PE) of genotypes was calculated based on the difference in dry forage yield of the fourth year (2019) from the second year (2017)^13^. Summer dormancy (S/SP) was calculated as the ratio of the summer yield of each genotype to the spring yield of the same genotype at normal irrigation condition by the following formula:

$${\text{S/SP}} = \left( {100 -\left[ {\left( {{\text{summer yield/spring yield}}} \right) \times 100} \right]} \right)/10.$$

The eight selection indices including stress susceptibility index (SSI)^48^, mean productivity (MP)^49^, stress tolerance (TOL)^50^, stress tolerance index (STI)^51^, geometric mean productivity (GMP)^52^, yield index (YI)^53^, yield stability index (YSI)^54^, and drought response index (DRI)^55^ for each genotype were calculated based on the dry forage yield (DFY) of normal, mild and intense drought stress conditions according to the following formulae:

2 $$SSI=\frac{1-({\text{Ysi}}/{\text{Ypi}})}{1-( \overline{Y}{\text{s}}/\overline{Y}{\text{p }})}$$

3 $$MP=(Ypi + Ysi)/2$$

4 $$TOL={\text{Ypi}}-{\text{Ysi}}$$

5 $$STI=\frac{{\text{Ypi }}\times {\text{ Ysi}}}{{(\overline{Y }p)}^{2}}$$

6 $$GMP=\sqrt{{\text{Ypi}}\times {\text{Ysi}}}$$

7 $$YI=\frac{{\text{Ysi}}}{\overline{Y}s }$$

8 $$YSI=\frac{{\text{Ysi}}}{{\text{Ypi}}}$$

9 $$DRI=\frac{{\text{Ysi}}-{\hat{\text{Y}}}\text{s}}{SE}$$

where Y_si_ is the total aerial biomass of the *i*th genotype under drought stress conditions and Y_pi_ is the potential yield of the *i*th genotype under normal environment. $\overline{Y }p$ and $\overline{Y }s$ are the mean yields of all genotypes under normal and drought stress conditions. The ${\hat{\text{Y}}}$s is the estimated potential yield for each genotype under drought stress conditions, and SE is the standard error of the estimated yield of all genotypes. ${\hat{\text{Y}}}$s is estimated for each specific genotype from a multivariate regression analysis based on the studied traits (only significant for Y_pi_, persistence (PE) and survival (SU) under both drought stress conditions) as follows:

10 $${\hat{\text{Y}}}{\text{s}}={\text{a}}+{\text{b}}({\text{Yp}})+{\text{c}}({\text{PE}})+{\text{e}}({\text{SU}})$$

where PE and SU are persistence and survival, and a, b, c, and e are regression parameters calculated by least square methods to estimate the value of ${\hat{\text{Y}}}$s. Finally, the following equation for stress tolerance score (STS) was proposed:

11 $$STS=MP+STI+GMP+YI+DRI+YSI-SSI-TOL-\upbeta$$

where β is the coefficient of linear regression in a model in which the forage yield of each genotype is the dependent variable and the environmental index was the independent variable. The environmental index is considered as the mean of all genotypes in each of the six environments (combination of two years and three irrigation levels).

Based on drought-tolerance/susceptibility equations, a large value for STI, MP, GMP, YI, DRI, and YSI, and a small value for SSI, TOL, and β represent relatively more tolerance to drought stress. Therefore, STI, MP, GMP, DRI, YI, and YSI had positive, and TOL, SSI, and β of indices had negative coefficients. To estimate a more accurate equation STS, all the indices of the above equation were standardized before calculating STS as follows:

12 $$Zij=\frac{{\text{Xij}}-\overline{X}{\text{i}}}{Si }$$

where *Zij* is the standard score for the *j*th genotype in the *i*th index, *Xij* is raw data of the *j*th genotype in the *i*th index, and *Si* is the standard deviation of the *i*th index^29^.

### Post-drought recovery

Post-drought recovery was assessed in all species after four years of evaluation in 2019. In the middle of August 2019, after the summer harvest, a long acute drought was imposed on all previous treatments (normal, mild, and intense drought stress) by stopping irrigation for 60 days (from 15 August to 15 October) until grass foliage was entirely desiccated (Fig. 5). Then all species were subsequently irrigated to the point of field capacity weekly to allow for prolonged drought stress recovery. To allow for prolonged drought stress recovery, all species were subsequently irrigated to the point of field capacity every week (Fig. 6). After almost 40 days (near the end of November) of regular re-watering, the recovery rate (RR) was measured. RR was visually scored based on the scale of 0 to 9, where genotypes with green and fully hydrated leaves were rated as 9, and desiccated brown/dead leaves were rated as 0.

### Statistical analysis

Data (residuals) were tested for normality using the Kolmogorov–Smirnov test, and homogeneity of variance was done with the Bartlett test. Analysis of variance (ANOVA) was performed using the Proc GLM in SAS 9.2^56^ to examine the differences between the environments, species, genotypes, and their interactions. Treatment means were compared using the least significant difference (LSD) test at P ≤ 0.05. Principal component analysis (PCA) was performed based on the correlation matrix, and biplots were drawn using Statgraphics software ver 17.2^57^.

## Supplementary Information

Supplementary Information.

## Data Availability

The datasets analyzed during the current study are available from the corresponding author on reasonable request.
