# Supplementary material for: Plant functional trait responses to cope with drought in seven cool-season grasses
Source: Sci Rep. 2023 Mar 31;13:5285. doi: 10.1038/s41598-023-31923-y (PMC10066319; doi:10.1038/s41598-023-31923-y)
Supplement: Supplementary file 1 — Supplementary Information. [file 41598_2023_31923_MOESM1_ESM.docx]

| Table S1. Information about plant materials and their origins used in this study | | | |
| --- | --- | --- | --- |
| Species | Code | Genotype/Variety | Origin |
| *Festuca arundinacea* |  |  |  |
|  | Fa1E | 1E | Iran, Isfahan |
|  | Fa21M | 21M | Iran, Isfahan |
|  | Fa17M | 17M | Iran, Isfahan |
|  | Fa4E | 4E | Iran, Isfahan |
|  | FaFe | Flecha | French, Institute INRA |
|  | FaLu | Lunibell | French, Institute INRA |
|  | FaBe | Belfine | Switzerland, Institute Agroscope |
|  | FaMo | Molva | Switzerland, Institute Agroscope |
|  | FaEl | Elfina | Switzerland, Institute Agroscope |
|  | FaBa | Barvado | Poland, Company Barenbrug |
| *Festuca pratensis* |  |  |  |
|  | FpPre | Prevel | Switzerland, Institute Agroscope |
|  | FpPra | Pradel | Switzerland, Institute Agroscope |
| *Festuca ovina* |  |  |  |
|  | FoOv69 | Ovina69 | Iran, Isfahan University of Technology |
|  | FoOv132 | Ovina132 | Iran, Isfahan University of Technology |
| *Festuca rubra* |  |  |  |
|  | FrRu1 | Rubra1 | Iran, Isfahan University of Technology |
|  | FrRu2 | Rubra2 | Iran, Isfahan University of Technology |
|  | FrRu3 | Rubra3 | Iran, Isfahan University of Technology |
|  | FrRu4 | Rubra4 | Iran, Isfahan University of Technology |
|  | FrRu5 | Rubra5 | Iran, Isfahan University of Technology |
|  | FrRu6 | Rubra6 | Iran, Isfahan University of Technology |
| *Lolium perenne* |  |  |  |
|  | LpArv | Arvela | Switzerland, Institute Agroscope |
|  | LpAri | Arion | Switzerland, Institute Agroscope |
| Lolium × hybridum |  |  |  |
|  | LhRu | Rusa | Switzerland, Institute Agroscope |
|  | LhRe | Redunca | Switzerland, Institute Agroscope |
|  | LhTa | Tapirus | Switzerland, Institute Agroscope |
| *Lolium multiflorum* |  |  |  |
|  | LmAl | Alces | Switzerland, Institute Agroscope |
|  | LmAx | Axis | Switzerland, Institute Agroscope |
|  | LmOr | Oryx | Switzerland, Institute Agroscope |

| Table S2. Analysis of variance for important traits in seven grass species (S) and 28 genotypes (G) subjected to three irrigation treatments (IT) (normal, mild and intense drought stress) | | | | |
| --- | --- | --- | --- | --- |
| S.V | df | Mean square | | |
|  |  | DFY | SU | RR |
| IT | 2 | 2663758^**^ | 13364^**^ | 462.41^**^ |
| Rep (IT) | 6 | 10604 | 15 | 1.98 |
| S | 6 | 1056417^**^ | 12318^**^ | 89.01^**^ |
| IT × S | 12 | 443672^**^ | 24118^**^ | 18.97^**^ |
| Rep (IT × S) | 36 | 5625 | 49 | 2.52 |
| G | 27 | 19707^**^ | 367^**^ | 0.96^**^ |
| IT × G | 54 | 11581^**^ | 105^**^ | 0.51^**^ |
| S × G | 162 | 12435^**^ | 163^**^ | 1.28^**^ |
| IT × S × G | 324 | 13271^**^ | 141^**^ | 0.98^**^ |
| Error | 1134 | 325 | 4 | 0.11 |
| CV |  | 12 | 5 | 9 |
| **: Significant at the 0.01 probability level  CV: coefficient of variation, df: degrees of freedom, Rep: replication, S.V: source of variation  DFY: dry forage yield, SU: survival, RR: recovery rate | | | | |

| Table S3. Analysis of variance for stress tolerance score (STS) in seven grass species (S) and 28 genotypes (G) subjected to two drought stress conditions (IT) (mild and intense drought stress conditions) | | |
| --- | --- | --- |
| S.V | df | Mean square |
|  |  | STS |
| IT | 1 | 33.54^**^ |
| Rep (IT) | 4 | 0.19 |
| S | 6 | 22.77^**^ |
| IT × S | 6 | 1.63^**^ |
| Rep (IT × S) | 24 | 0.26 |
| G | 27 | 4.82^**^ |
| IT × G | 27 | 2.11^**^ |
| S × G | 162 | 2.34^**^ |
| IT × S × G | 162 | 0.71^**^ |
| Error | 756 | 0.19 |
| CV |  | 11 |
| **: Significant at the 0.01 probability level  CV: coefficient of variation, df: degrees of freedom, Rep: replication, S.V: source of variation | | |

| Table S4. Analysis of variance for persistence (PE) and summer dormancy (S/SP) in seven grass species (S) and 28 genotypes (G) subjected to normal irrigation condition | | | |
| --- | --- | --- | --- |
| S.V | df | Mean square | |
|  |  | PE | S/SP |
| Rep | 2 | 0.30^ns^ | 0.20^ns^ |
| S | 6 | 740731.81^**^ | 16.77^**^ |
| Rep × S | 12 | 6.16 | 0.52 |
| G | 27 | 19177.55^**^ | 1.78^**^ |
| S × G | 162 | 18881.96^**^ | 1.77^**^ |
| Error | 378 | 6 | 0.47 |
| CV |  | 18 | 15 |
| **, and ns: Significant at the 0.01 probability level and non-significant  CV: coefficient of variation, df: degrees of freedom, Rep: replication, S.V: source of variation | | | |

| a) Mild drought stress | b) Intense drought stress |
| --- | --- |
|  |  |
|  |  |
| Figure 1S. Biplot of stress tolerance score (STS) vs. persistence (PE) and recovery rate (RR) for 28 genotypes belonged to seven species under two drought stress levels (mild and intense drought stress) | |
